# Drug-Induced Parkinsonism or Parkinson’s Disease: A Diagnostic Challenge in a Psychiatric Patient

**DOI:** 10.7759/cureus.88177

**Published:** 2025-07-17

**Authors:** Wiktoria Balcerzak, Agnieszka Gorzkowska, Marta Konieczna, Anna Blach, Anetta Lasek-Bal

**Affiliations:** 1 Department of Neurology, Upper-Silesian Medical Center, Medical University of Silesia, Katowice, POL; 2 Department of Neurology, School of Health Sciences, Medical University of Silesia, Katowice, POL; 3 Department of Cardiology and Structural Heart Diseases, Medical University of Silesia, Katowice, POL; 4 Department of Nuclear Medicine, Voxel Diagnostic Center, Katowice, POL

**Keywords:** datscan, drug-induced parkinsonism, neuroleptics, parkinson' s disease, schizophrenia

## Abstract

Drug-induced parkinsonism (DIP) is the second most common cause of parkinsonian syndromes after idiopathic Parkinson’s disease (iPD), accounting for approximately 15-25% of cases in older adults. While DIP typically results from antipsychotic-induced dopamine receptor blockade and is often reversible, iPD is a progressive neurodegenerative disorder characterized by asymmetrical onset, resting tremor, and good response to levodopa. Distinguishing between these conditions is clinically important but often challenging. The aim of this article is to discuss the diagnostic and therapeutic challenges involved in differentiating DIP from iPD in patients with psychiatric disorders who are chronically treated with antipsychotics. Based on the case of a 52-year-old man with a long-standing history of schizophrenia treatment, the diagnostic process and clinical manifestations leading to the final diagnosis are presented. The patient developed Parkinsonian symptoms during olanzapine therapy, initially suggesting DIP. This paper discusses the clinical indicators differentiating DIP from iPD, as well as the critical role of single photon emission computed tomography (SPECT) with ioflupane-123 (DaTSCAN) in the differential diagnosis of Parkinsonism in selected, ambiguous cases. The described case illustrates the phenomenon of so-called "unmasked Parkinson’s disease" and highlights the need for detailed neurological monitoring in patients suspected of having DIP. It is essential to consider iPD as an independent cause of movement disorders in patients treated with antipsychotics. Early diagnosis and appropriate treatment of the underlying cause of Parkinsonism can significantly reduce extrapyramidal symptoms and improve the patient’s quality of life.

## Introduction

Drug-induced parkinsonism (DIP) is the second most common cause of parkinsonian syndromes in the elderly, after idiopathic Parkinson’s disease (iPD). DIP accounts for approximately 15-25% of all Parkinsonism cases in older adults. It most frequently develops in patients receiving treatment with dopamine D2 receptor antagonists, particularly antipsychotics (e.g., first-generation agents such as haloperidol and chlorpromazine and second-generation agents such as risperidone and olanzapine). By contrast, idiopathic Parkinson’s disease has a prevalence of approximately 1-2% in individuals over 60 years of age and an incidence of about 10-18 per 100,000 person-years [1,2]. Elderly individuals, women, and those using high-potency first-generation antipsychotics are especially at risk [1].

DIP symptoms typically emerge within a few weeks of initiating pharmacotherapy or increasing the medication dose. In most cases, symptoms resolve after discontinuation of the causative agent. However, in some patients, Parkinsonian symptoms persist despite stopping or reducing the antipsychotics. In such cases, it may sometimes be interpreted as the manifestation of previously undiagnosed, subclinical iPD unmasked by the drug [2].

Idiopathic Parkinson’s disease is a progressive neurodegenerative disorder characterized by asymmetric bradykinesia, rigidity, resting tremor, and postural instability. Unlike DIP, iPD often begins insidiously, with motor symptoms developing slowly over the years, and is frequently accompanied by non-motor prodromal features such as hyposmia, rapid eye movement (REM) sleep behavior disorder, depression, and autonomic dysfunction.

A key differentiating factor between DIP and iPD is presynaptic dopaminergic integrity, which can be evaluated using dopamine transporter SPECT imaging (DaTSCAN). In DIP, DaTSCAN typically shows normal striatal uptake, indicating preserved nigrostriatal neurons. In contrast, iPD shows reduced and often asymmetric tracer uptake, reflecting underlying neurodegeneration.

Cohort studies indicate that individuals diagnosed with DIP have a significantly increased risk of developing iPD in the future, suggesting that DIP may represent one of the potential prodromal presentations of PD [3]. Consequently, patients with DIP should remain under long-term neurological observation, even after the resolution of initial symptoms, with particular attention paid to early signs of developing iPD. Additionally, clinicians should consider not only the current clinical presentation but also each patient’s individual risk factors and the potential for future development of full-blown iPD.

The diagnostic distinction between DIP and iPD is particularly important in patients with chronic psychotic disorders because dopaminergic treatment of Parkinsonism risks exacerbating psychosis. Accurate diagnosis guides appropriate therapy and helps balance motor symptom control with psychiatric stability. Initiating dopaminergic therapy in patients with chronic psychotic disorders is inherently risky due to the potential exacerbation of psychosis, requiring close psychiatric monitoring and interdisciplinary management.

This article presents the case of a patient who developed Parkinsonian symptoms during long-term psychiatric treatment. The case required an in-depth diagnostic evaluation to differentiate iPD from DIP and to initiate appropriate symptomatic therapy.

## Case presentation

A 52-year-old male patient with a 25-year history of treatment for schizophrenia was admitted to the Neurology Department for a planned short diagnostic hospitalization lasting three days. He was referred by his treating psychiatrist due to progressive muscle rigidity, predominantly in the lower limbs (more pronounced on the left side), bradykinesia, and worsening balance, with increasingly frequent falls over the past four years. The patient's psychiatric history included chronic schizophrenia with longstanding symptoms such as persecutory delusions and occasional auditory hallucinations, which had remained stable for many years on the current medication regimen. He reported that symptoms were most severe in the morning, with partial improvement as the day progressed. Importantly, he did not report any prodromal non-motor symptoms of Parkinson’s disease, such as a diminished sense of smell, constipation, dream-enactment behaviors during sleep (REM sleep behavior disorder), or other autonomic disturbances, prior to the onset of his movement problems. There was no known history of substance abuse, head trauma, or relevant family history of movement disorders. No additional social or occupational history was documented as directly relevant to the differential diagnosis.

Over the last 10 years, the patient had been taking a second-generation antipsychotic, olanzapine, at 10 mg once daily, along with mianserin 60 mg once daily (no reliable information was available regarding earlier antipsychotic treatments or doses). Due to progressive complaints of left-sided limb rigidity, bradykinesia, balance difficulties, and increasingly frequent falls, the patient had been started on oral levodopa treatment, a dopamine agonist, and biperiden six months ago. These symptoms progressively impacted his daily activities and increased his dependence on others for mobility and self-care. At the time of admission, the daily doses of the medications were as follows: levodopa 600 mg in three doses, ropinirole 8 mg once a day, and biperiden 4 mg once a day (levodopa equivalent daily dose, LEDD: 960 mg). The patient’s psychiatric regimen (olanzapine and mianserin) had remained unchanged. Notably, his mental state remained stable throughout the introduction of dopaminergic therapy (based on clinical assessment, as no standardized psychiatric rating scales were available), and he had experienced partial improvement in his Parkinsonian motor symptoms with this treatment.

Neurological examination revealed the following: the patient was alert and oriented to person, place, and time but psychomotorically slowed, with hypomimia, dysarthric speech, axial rigidity, peripheral left-sided facial nerve paresis, increased extrapyramidal muscle tone in all four limbs, bradykinesia with left-sided predominance, resting and postural tremor in the left upper limb, propulsion and lateropulsion with a tendency to fall to the left, and a parkinsonian gait with a stooped posture and reduced arm swing. A levodopa challenge test (LCT) was performed following a standard protocol. The patient was kept in an overnight OFF state (withdrawal of dopaminergic medication for at least 12 hours) before the test. Baseline motor assessment was conducted using the UPDRS part III, with an OFF score of 52 points. The patient then received 200 mg of soluble levodopa, and motor symptoms were reassessed 60 minutes post-dose, improving to 37 points, indicating a 30% improvement. As this met the standard threshold for a positive response, repeat testing was not pursued. Mental status was clinically assessed and found to be stable, without signs of acute psychosis or mood disorder, though no formal psychometric scales were applied. Laboratory investigations, including routine hematology and biochemistry panels, were unremarkable. Vital signs were stable throughout admission with no significant abnormalities. A consultation with a speech therapist revealed hypokinetic dysarthria, with no evidence of dysphagia.

Brain MRI showed two minor findings: vascular-degenerative changes and an arachnoid granulation in the right transverse sinus, measuring approximately 12 × 9 mm.

**Figure 1:**
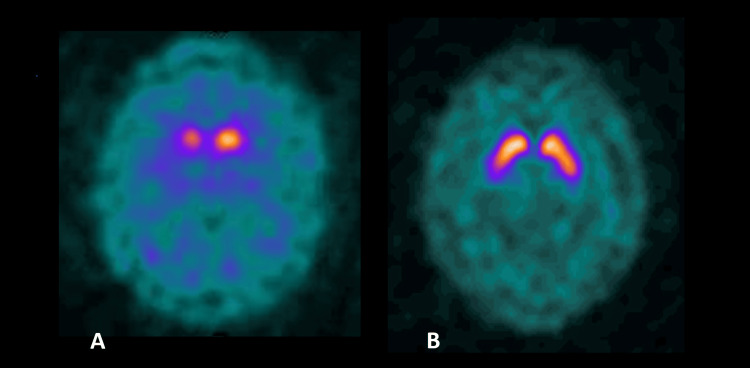
A. A 52-year-old patient’s DaTSCAN SPECT image showing significantly reduced, asymmetric uptake of 123I-ioflupane in the striatum. Lack of tracer accumulation bilaterally in the putamen and asymmetry of tracer uptake in the caudate nuclei with significantly lower activity on the right side. Abnormal increased tracer accumulation in the background. B. Normal DaTSCAN SPECT reference image showing normal uptake in the striatum and background. DaTSCAN: Dopamine transporter scan; SPECT: Single photon emission computed tomography The attached images are fully diagnostic images of this type of examination. The single photon emission computed tomography (SPECT) method is characterized by significantly lower resolution than other imaging methods, such as CT or MRI, and the scans obtained have a ‘blurred’ component. A sharper image would require digital processing, which would not be consistent with the nature of this examination.

It is important to note that treatment strategies for DIP and iPD differ fundamentally, especially in the context of a comorbid psychiatric condition. [2] Patients with schizophrenia who are treated with dopaminergic replacement therapy are at risk for exacerbation of psychiatric symptoms. Therefore, it was decided to extend the diagnostic workup with a DaTSCAN before increasing the levodopa dose. The DaTSCAN results were abnormal, showing bilateral reduced dopamine transporter activity with right-sided predominance (Figure 1). Based on these findings, a diagnosis of idiopathic Parkinson’s disease was established.

Following the confirmation of iPD, the patient’s antiparkinsonian regimen was adjusted. The levodopa daily dose was gradually increased to 1000 mg in four divided doses (250 mg each), while the ropinirole dose was reduced from 8 mg once daily to 4 mg once daily as part of the withdrawal plan, with further tapering planned to minimize potential neuropsychiatric side effects.

At the six-month follow-up visit, conducted as a planned single-day diagnostic admission, the patient’s antiparkinsonian regimen included levodopa 1000 mg daily in four divided doses (250 mg each) and biperiden 4 mg once daily. Ropinirole, which had been gradually tapered in the preceding months, was fully discontinued at this visit. Antipsychotic treatment was continued without change (olanzapine 10 mg/day and mianserin 60 mg/day). The patient showed sustained improvement in bradykinesia and rigidity, no longer experienced falls, and reported better overall mobility and independence in daily activities. His mental state remained stable without psychotic exacerbation. The patient’s quality of life improved notably owing to symptom control and diagnostic clarity. No new therapies were initiated at this time, and continued neurological and psychiatric follow-up was recommended. This case also involved interdisciplinary collaboration between neurology and psychiatry. Treatment planning and diagnostic evaluation were coordinated with the patient’s treating psychiatrist to balance motor symptom management with psychiatric stability.

## Discussion

Differentiating between DIP and iPD is a diagnostic challenge that requires a thorough analysis of the clinical picture, along with additional testing. Important features include timing of onset, symmetry of motor signs, resting tremors, response to medication changes or withdrawal, levodopa responsiveness, and imaging such as DaTSCAN. 

Key clinical features that help differentiate DIP from iPD include: 1) Timing: DIP develops within weeks of starting or increasing antipsychotic dose; iPD has an insidious, gradual onset. 2) Symmetry: DIP is usually bilateral and symmetric; iPD often starts asymmetrically. 3) Resting tremor: Rare or mild in DIP; common and unilateral at onset in iPD. 4) Response to drug withdrawal: DIP often improves or resolves after reducing or stopping antipsychotics; iPD does not remit spontaneously. 5) Levodopa responsiveness: Typically poor in DIP; robust improvement in iPD. 6) DaTSCAN: Dopamine transporter SPECT imaging is generally normal in DIP (indicating preserved presynaptic dopaminergic neurons), while in iPD it is abnormal, showing reduced striatal uptake (often with an asymmetric pattern corresponding to the more affected side).

A structured comparison of these key features is provided in Table 1 [4-6].

**Table 1:** Diagnostic criteria and key differentiating features of drug-induced parkinsonism versus idiopathic Parkinson’s disease. iPD: Idiopathic Parkinson’s disease; DIP: Drug-induced parkinsonism; DaTSCAN: Dopamine transporter scan. Adapted from [4-6]

Criterion	DIP	iPD
Association with medication	Clear temporal relationship with exposure to dopamine-blocking agents (e.g., antipsychotics). Symptoms typically develop within weeks to months of starting or increasing dose.	No association with medication use. Onset is insidious and spontaneous, with no identifiable pharmacologic trigger.
Symmetry of symptoms	Usually bilateral or only mildly asymmetric.	Typically asymmetric, often starting on one side of the body.
Rest tremor	Rare or mild if present; often symmetric.	Common, prominent, usually unilateral at onset.
Non-motor symptoms	Typically, absent. Lacks prodromal features such as hyposmia, REM sleep behavior disorder, or autonomic dysfunction.	Frequently present, including hyposmia, REM sleep behavior disorder, constipation, depression, and other autonomic symptoms.
Course after drug withdrawal	Symptoms often improve or resolve within weeks to months after reducing or stopping the offending medication (though ~20% may have persistent symptoms).	Chronic, progressive course independent of any drug withdrawal. Symptoms do not remit spontaneously.
Response to levodopa	Typically, minimal or absent.	Usually robust improvement (>30% in levodopa challenge test).
DaTSCAN imaging	Normal dopamine transporter uptake (preserved presynaptic function).	Abnormal, showing reduced and often asymmetric striatal uptake.
Additional features	May coexist with other drug-induced movement disorders (e.g., akathisia, dystonia, tardive dyskinesia).	Classic parkinsonian features without medication-induced movement disorders (though levodopa-induced dyskinesias may develop over time).
Diagnostic criteria	Presence of parkinsonism (bradykinesia plus tremor and/or rigidity); clear temporal relationship to dopamine-blocking medication; no prior parkinsonian signs; improvement with drug reduction or withdrawal (if possible); exclusion of other causes.	Parkinsonism (bradykinesia plus tremor and/or rigidity); exclusion of alternative causes (including DIP); supportive criteria: unilateral onset, progressive course, clear levodopa response, levodopa-induced dyskinesias, resting tremor; no absolute exclusion criteria (e.g., normal DaTSCAN).

Additionally, it is important to consider the formal diagnostic criteria. DIP is defined by Parkinsonism with a clear temporal relationship to dopamine-blocking medication use, no prior Parkinsonian signs, and improvement after drug reduction or withdrawal. By contrast, iPD is diagnosed by Parkinsonism without a secondary cause, often with asymmetric onset, a progressive course, and a clear, sustained response to levodopa therapy.

DIP most commonly presents with a symmetrical distribution of motor symptoms, typically affecting the upper limbs. In contrast, motor symptoms in iPD patients, especially in the early stages, are often clearly asymmetrical, which is a significant differentiating feature. Resting tremors, a hallmark of iPD, are less common and usually milder in DIP. The clinical picture of DIP is predominantly characterized by muscle rigidity, bradykinesia, and various extrapyramidal signs, including dystonia, dyskinesia, and akathisia. Changes in speech articulation are also frequently observed [7,8].

In contrast, resting tremors play a significant diagnostic role in iPD. The clinical course of iPD is characterized by gradual progression and the presence of non-motor symptoms such as olfactory disturbances, depressive symptoms, REM sleep behavior disorder, and signs of autonomic dysfunction, all of which may suggest a neurodegenerative etiology [8].

In our patient, the clinical picture alone was equivocal. He presented with bilateral Parkinsonian signs, but there was notable asymmetry (left-sided predominance of bradykinesia, rigidity, and tremor) and the presence of a resting tremor, features more suggestive of iPD. On the other hand, he lacked some features often associated with idiopathic PD (such as any non-motor symptoms or a strictly unilateral onset), and his parkinsonism developed in the context of ongoing antipsychotic therapy, which initially pointed toward DIP. These mixed clinical features were not sufficient to definitively differentiate between DIP and iPD based on history and examination alone.

Several findings in this case ultimately pointed toward a diagnosis of idiopathic Parkinson’s disease rather than drug-induced Parkinsonism: 1) Gradual progression of symptoms over four years, without any recent change in antipsychotic dose. 2) Asymmetry of motor signs, with left-sided bradykinesia, rigidity, and resting tremor. 3) Partial but significant response to levodopa (30% improvement on challenge test). 4) Abnormal DaTSCAN with asymmetric reduced striatal uptake, confirming presynaptic dopaminergic neuron loss.

The temporal relationship and response to treatment modification are significant factors in differentiating between DIP and iPD. DIP typically develops within a short timeframe, usually a few days to several weeks, after the initiation or dose increase of an antipsychotic. In most cases, symptoms resolve after discontinuation of the drug, supporting a direct causal link (approximately two-thirds of patients recover within seven weeks, though some may take up to 18 months) [2]. In contrast, iPD follows a chronic, progressive course that is independent of pharmacotherapy [9].

Another key differentiating factor is the clinical response to levodopa treatment. In iPD, a notable improvement in motor function is typically observed, while in DIP, the effect is generally minimal or absent. Due to the risk of exacerbating psychotic symptoms, LCT should be performed cautiously and only in carefully selected patients [2]. In this case, withdrawal of the antipsychotic was not possible due to the patient's psychiatric condition. Additionally, while quetiapine can cause extrapyramidal symptoms, it carries a significantly lower risk compared to typical antipsychotics [2]. The result of the LCT (<50% improvement) was inconclusive, and as such, the assessment of Parkinsonian symptoms in response to pharmacotherapy did not further support the diagnostic process.

A particularly valuable diagnostic tool in this context is DaTSCAN. In DIP, striatal tracer uptake is typically normal, indicating preserved presynaptic neuronal function. In iPD, a significant reduction in signal is observed, reflecting the neurodegenerative nature of the disease. However, it is important to note that some DIP patients may initially show normal DaTSCAN results, which may later evolve to reveal reduced uptake, suggesting a subclinical form of iPD [10]. In other words, an initially normal scan in a patient with presumed DIP doesn’t completely rule out the possibility of underlying neurodegeneration; it may simply mean the disease process has not advanced enough to be detected at that time. In the presented case, DaTSCAN was the decisive test. Obtaining a DaTSCAN is especially useful in challenging cases like this one, where clinical features are mixed or confounded by medication effects. In fact, one of the primary indications for DaTSCAN imaging is to help differentiate DIP from iPD when the diagnosis is uncertain. That said, DaTSCAN should be used judiciously, reserved for scenarios in which the diagnosis remains unclear after thorough clinical evaluation, given its cost and limited availability [10].

In clinical practice, careful analysis of the patient’s history, symptom distribution, onset dynamics, and the presence of non-motor signs may not always enable an accurate distinction between DIP and iPD. In such cases, dopamine system imaging should be employed to guide appropriate diagnostic and therapeutic management. The authors would like to emphasize that it is critical to consider less obvious causes of movement disorders in these patients, as this can initiate the diagnostic process.

Additionally, in real-world clinical settings, neurologists should not only consider the current clinical presentation and the results of ancillary tests but also take into account individual risk factors and the potential for future development of full-blown iPD. For this reason, the use of prodromal Parkinson’s disease criteria, as proposed by the Movement Disorder Society (MDS), is recommended to assess the risk of idiopathic disease emergence in patients diagnosed with DIP. These research criteria aim to estimate the probability that an individual is in the early neurodegenerative stage before classic motor symptoms fully develop. They incorporate multiple risk and prodromal markers, including non-motor features (such as REM sleep behavior disorder, hyposmia, constipation, and depression), subtle motor signs, abnormal DaTSCAN findings, and risk factors like family history or environmental exposures (e.g., pesticides). This probabilistic approach supports early recognition and monitoring in high-risk individuals [4].

From a therapeutic perspective, this case underscores the substantial challenge of managing Parkinsonian symptoms in a patient with a chronic psychotic disorder. The pharmacological treatments for iPD and for schizophrenia have opposite effects on the dopamine system: treating Parkinson’s disease requires enhancing dopaminergic activity (e.g., with levodopa or dopamine agonists), whereas treating schizophrenia requires suppressing dopaminergic activity (e.g., with D2-receptor-blocking antipsychotics). This fundamental conflict means that attempts to improve Parkinsonian symptoms with dopaminergic drugs can potentially destabilize the patient’s psychiatric condition, while reducing or discontinuing antipsychotic medication to alleviate Parkinsonism can risk a psychotic relapse. Effective management, therefore, demands a delicate balance and close collaboration between neurologists and psychiatrists. Formal psychiatric rating scales were not performed in this case, but the patient’s mental state remained stable throughout dopaminergic therapy. One limitation in our management approach was the lack of formal psychiatric rating scale assessments (such as the Positive and Negative Syndrome Scale [PANSS] or Brief Psychiatric Rating Scale [BPRS]) to quantitatively monitor the patient’s psychotic symptoms over the course of treatment.

In patients where diagnostic findings support a diagnosis of iPD, management should follow standard PD treatment protocols. Treatment must address both the motor symptoms of iPD and any coexisting psychiatric disorders. The cornerstone of iPD treatment is dopaminergic therapy, most commonly levodopa combined with a decarboxylase inhibitor, such as levodopa/benserazide or levodopa/carbidopa. Treatment should begin with the lowest effective dose, gradually increasing based on the patient's clinical response [11].

In patients diagnosed with schizophrenia or other psychotic disorders, dosing should be particularly cautious. Treatment should start at low doses and be increased slowly to minimize the risk of triggering or exacerbating psychotic symptoms [12,13]. Close psychiatric monitoring is essential to detect any early signs of psychosis exacerbation during dopaminergic therapy. Alternative dopaminergic options, such as dopamine agonists (DAs), including ropinirole, pramipexole, and rotigotine, are often used, but their adverse effects profile limits their use in patients with psychiatric comorbidities [14,15]. Studies have shown that amantadine is associated with an increased risk of psychotic symptoms, such as hallucinations and delusions, and therefore is not recommended for patients with iPD and a history of psychiatric illness [16].

In the case of the diagnosed patient, levodopa was initiated following suspicion of iPD, and the dose was gradually increased. Ropinirole was initially introduced, but, recognizing its higher propensity to induce hallucinations and other neuropsychiatric side effects, it was carefully reduced and eventually withdrawn to mitigate the risk of psychiatric complications. Biperiden, while helpful in alleviating both DIP and iPD symptoms, should be used cautiously in patients with psychiatric symptoms, as in this case, because anticholinergic agents can cause central nervous system side effects such as confusion, hallucinations, and cognitive impairment that may exacerbate psychiatric conditions [11].

For patients with a history of psychotic episodes, dopaminergic therapy should be combined with atypical antipsychotics that have low D2 receptor blockade potential, primarily quetiapine or clozapine. Clozapine, despite requiring regular blood count monitoring due to the risk of agranulocytosis, is considered the most effective antipsychotic for treating psychosis in PD patients, as it has minimal impact on motor symptoms. If our patient had shown signs of worsening psychosis, switching to clozapine with appropriate hematological monitoring would have been a preferred option. Quetiapine, on the other hand, tends to be better tolerated but has limited efficacy in managing psychotic symptoms [2,13,17]. In the case of our patient, who was treated with olanzapine, we recommend continuing this therapeutic option, given its benefits in managing psychiatric symptoms.

The primary therapeutic strategy for managing DIP is modifying the existing pharmacotherapy, particularly the antipsychotic medications. In collaboration with the treating psychiatrist, efforts should be made to reduce the dose of the causative drug or, if possible, discontinue it altogether. This should lead to gradual clinical remission of the Parkinsonian symptoms [18]. However, it is important to note that for medications with long half-lives, improvement may be delayed and can take several months. Furthermore, it is estimated that in approximately 20% of patients, Parkinsonian symptoms persist despite drug withdrawal or may even worsen over time [2].

The treatment of iPD should be comprehensive, encompassing both pharmacological and non-pharmacological interventions to improve the quality of life for patients and their caregivers. Key elements of this approach include physical rehabilitation, occupational therapy, psychological support, and education for both the patient and their caregivers. These multidisciplinary interventions not only address motor and non-motor symptoms but also help maintain patient independence, reduce caregiver burden, and improve overall quality of life, underscoring the need for integrated, patient-centered care models.

## Conclusions

This case underscores the diagnostic and therapeutic challenges associated with Parkinsonian syndromes in patients receiving long-term antipsychotic treatment. While DIP often resolves after withdrawal of the causative agent, symptoms may persist or progress, raising suspicion of iPD unmasked by antipsychotic use. Accurate diagnosis requires a comprehensive assessment of the clinical course, including evaluation for non-motor symptoms when present, response to levodopa therapy, and, when clinical features remain ambiguous, dopamine transporter SPECT imaging, which can provide objective evidence in selected cases and help support diagnostic confidence. Patients with DIP also remain at increased risk of developing iPD, underscoring the need for long-term neurological follow-up even after apparent symptom resolution.

When iPD is confirmed, treatment should follow established guidelines with careful adjustments for the psychiatric context, including close psychiatric monitoring, ideally with formal rating scales when feasible, to minimize the risk of exacerbating psychosis. This emphasizes close interdisciplinary collaboration between neurology and psychiatry to balance dopaminergic therapy and psychiatric stability. Early implementation of appropriate treatment can significantly improve motor function and quality of life. Looking ahead, interdisciplinary management involving neurologists, psychiatrists, and rehabilitation specialists should become the standard of care to enable early diagnosis, personalized treatment based on individual risk profiles, and ultimately, better patient outcomes.
